# Parent Perceptions of an Anxiety Prevention Manual for Young Children

**DOI:** 10.3390/ijerph22060833

**Published:** 2025-05-26

**Authors:** Olutosin Sanyaolu, Ava Robertson, Tabitha Naa Akuyea Addy, Laura Anne Nabors

**Affiliations:** 1 Public Health Department, School of Arts and Sciences, Fort Lewis College, Durango, CO 81301, USA; osanyaolu@fortlewis.edu; 2 Department of Psychology, College of Arts and Sciences, University of Cincinnati, Cincinnati, Oh 45221, USA; rober3av@mail.uc.edu; 3 School of Human Services, College of Education, Criminal Justice, Human Services, and Information Technology, University of Cincinnati, Cincinnati, Oh 45221, USA; addytn@mail.uc.edu

**Keywords:** anxiety prevention, young children, anxiety management, cognitive-behavioral strategies, emotional functioning

## Abstract

Parents are primary “supporters” for helping their children cope with feelings of anxiety, a significant concern for many young children. The current study examined parents’ perceptions of an anxiety management manual. Parents reviewed an anxiety coping manual for elementary school-aged children. This manual explained how anxiety influences the body and emotions, as well as presenting cognitive-behavioral anxiety management strategies. The strategies included breathing, imagery (superhero to fight worries and imagine your favorite place), relaxation, talking to supportive others, and using distraction. Convenience samples of 15 parents completed virtual interviews and 6 completed in-person interviews to determine their perceptions of the manual and of worry for today’s children. Qualitative analyses were performed to determine themes in the data. Results indicated that parents would use the manual and key themes, which were (1) learning new strategies for helping their child, (2) discussing children’s worries, and (3) sharing why the worry strategies would be useful (e.g., for emotion regulation). Parents felt that today’s children are worrying more about serious things like school performance and family stressors. Future research needs to examine parent implementation of the strategies over time to determine if the use of anxiety management strategies is related to lower levels of worry for young children, if the strategies reduce anxiety-related stress, and if prevention minimizes the impact of anxiety on emotional functioning.

## 1. Introduction

Anxiety is a normal response to stressful experiences, and it is common in children [1,2]. Muris et al. (2000) [3] found that over 70% of children between four and twelve years of age reported some experience of anxiety. Children worry about many things, including school performance, friendships, negative social experiences (like teasing), becoming ill or even dying [4]. Anxiety may be defined as, “…emotional reactions arising from …(thinking about, anticipating)… a threat to the self” (p. 25) [5]. Wehry et al. (2015) [6] found that about 15–20% of children experience anxiety at some point in their lives. Rates vary across studies, and some show lower prevalence rates, about 8% for anxiety disorders in children, and anxiety is typically more common in girls than boys [7]. If anxiety is not addressed, symptoms may persist into later years (i.e., adulthood) [8,9]. Prevention strategies or learning coping strategies in the early years may help children cope with feelings of anxiety, which, in turn, sets a positive trajectory for moving through anxiety-provoking situations, improves coping skills, and sets a positive trajectory for being able to cope with worrisome situations. Moreover, anxiety can run “in the family”, so it is important to teach parents coping strategies so that they can teach their children and role model positive coping [10].

Children need to learn about how anxiety affects both the body and mind, as well as learn coping strategies, and parents who know these things are teachers to improve their children’s abilities to cope with anxious feelings [11,12]. Along with learning coping strategies, young children need to know that anxiety or “worry” about different situations is part of life, and they need to understand how anxiety impacts them physically and mentally [11,13]. Children may experience changes in breathing, heart rate, nausea, and perspiration rate. They may shake or cry. Children also need to understand rumination, which is reflecting on something that makes them anxious, so that they avoid coping with their stressors [2]. Children also need coping skills, such as cognitive-behavioral strategies (breathing, positive imagery, thinking, distraction, and relaxation), to cope with their worries, de-stress, and continue with their daily routines [12,13,14,15]. Parents may not seek therapy services or interventions to help children with anxiety [16], and, as such, having prevention-oriented materials for parents may assist them in helping their children learn to cope.

Evidence-based, prevention-oriented materials that adopt strategies such as cognitive-behavioral coping techniques have proven effective in managing anxiety [17]. These practical strategies, including deep breathing exercises, positive self-talk, distraction techniques, mindfulness activities, muscle relaxation, and guided imagery, foster anxiety management among children, helping them stay calm in moments of distress and build emotional resilience [14,18]. For instance, deep breathing exercises help regulate the nervous system [19]. Additionally, positive self-talk and cognitive restructuring encourage children to challenge worrying thoughts and replace them with more balanced perspectives to reduce worry intensity [20]. Further, distraction techniques, such as engaging in hobbies, playing with games or toys, movement-based activities, or sensory grounding, redirect children’s attention from distressing thoughts and promote emotional regulation [21]. Muscle relaxation and guided imagery techniques help children recognize body tension and actively practice relaxation [18].

Parents can support children with effective coping mechanisms that enhance their ability to navigate stress and anxiety by incorporating these cognitive-behavioral strategies into daily routines [22]. These foundational skills can be reinforced at home, helping children develop long-term emotional resilience before professional intervention is needed [23]. Studies have shown that involving parents in anxiety interventions for children effectively reduces their children’s anxiety and fosters long-term emotional resilience [22,24]. Specifically, training parents equips them with skills to reinforce using strategies at home, creating a consistent support system that improves the child’s ability to internalize coping mechanisms and reduce anxiety over time [22]. Parents’ involvement strengthens parent-child relationships, helps them understand the child’s experiences, and promotes a supportive environment where they model effective coping mechanisms themselves, further promoting a positive and adaptive approach to stress management within the family [23].

This pilot study examined parent perceptions of a children’s book, “*Coping Positively with My Worries Book*”, a children’s manual or workbook featuring cognitive-behavioral strategies for anxiety management for elementary school-age children [25]. Data were collected through virtual and in-person interviews. The virtual interviews were conducted first, and then a second phase of the study, with in-person meetings, using the same interview questions, was used as a verification check for the information provided by parents during virtual interviews [26]. Parents provided information about whether the book would be useful for their children, as well as providing their opinions about the strategies in the book, such as which strategies they might use with their child. Information about the strategies parents used before the review of the book, as well as their perceptions of worry in today’s children, was also assessed.

This pilot study sought to examine parents’ perceptions of a children’s manual to determine the need for the manual and evaluate its usefulness. It was expected that parents would report that some of the strategies were “new” to them, showing a need for the intervention. It was hypothesized that parents would report that their children were experiencing worry about real-world problems, such as social media. It was anticipated that parents would report they would use the strategies in the manual to help their child decrease feelings of anxiety. Results provide information on parent perspectives related to how they perceive and plan to cope with worries for their young children, which is an important aspect of the study, as prevention of anxiety in children will improve their emotional coping and has the potential to prevent more significant experiences of anxiety for children.

## 2. Materials and Methods

### 2.1. Instrumentation

*Coping Positively with My Worries Book*. This manual is evidence-based, focusing on cognitive-behavioral strategies that will work to help prevent anxiety and help as an intervention for those children experiencing anxiety [13,25]. The manual, which was developed as a children’s workbook/book, begins with a developmentally appropriate explanation of how worry works in the body and what it can feel like. Then, there are six worry management strategies covered in the workbook, including relaxation (breathing and muscle relaxation), positive imagery, distraction, talking to someone else, using positive self-talk, and challenging worry using your own superhero. The workbook has a developmentally appropriate explanation of how to use each worry management strategy, and there are instructions for practicing the strategy with the child (see Nabors et al. supplementary material for the manual or workbook [25]). At the end of the workbook, there is a coping menu where children can list their favorite strategies.

Interview. Parents provided demographic information on their sex, age range, occupation, and they provided demographic information about their child (e.g., sex, age, and grade). Then, parents completed questions to assess their perceptions of the worry manual. These questions addressed their perceptions of the manual, what they thought of the strategies, what strategies they were using before reviewing the manual, what new strategies they learned, and their ideas of strategies they would use (or not use) as well as when/how they would implement anxiety management strategies [25]. They also responded to a question about whether they thought today’s children were worrying and what they were worrying about [25].

### 2.2. Participants

Twenty-one parents participated in interviews. Fifteen parents completed virtual interviews, and six completed in-person interviews. Participants were from Ohio, Kentucky, Georgia, Alabama, and Colorado. A university-based institutional review board approved this study.

### 2.3. Procedures

Procedures for Virtual Interviews. Parents were referred to L. N. by students, and a recruitment flier was placed on a local parent’s website. Snowball sampling was used to recruit additional parents. Parents received an email from L. N. explaining the study with a consent form. If they elected to participate, they emailed written consent for study participation to L. N. Participants reviewed the children’s manual. Then, they completed interviews to assess their opinions of the manual via meetings on Microsoft TEAMS or Zoom. The interviewers were L. N. and T. N. A. Parents completed the interview questions, and probes were: tell me more about it, provide an example, and please explain what you mean. Interviews lasted between 15 and 45 min, with an average interview lasting about 30 min. Interviewers reviewed and transcribed the recordings and also used their notes from the meetings to develop transcripts in Microsoft Word.

Procedures for In-Person Interviews. Parents were volunteers from two elementary schools in Ohio who agreed to complete an interview when they were participating in a parent-teacher night to learn of their child’s school progress. Parents completed a consent form. They were interviewed by L.N. at two schools, three parents at each school. The same interview questions and probes were used from Study 1. Interviews lasted between 15 to 30 min, with an average of about 20 min. L.N. reviewed her notes from each meeting and typed them into a Microsoft Word document to develop transcripts.

Approach. An inductive approach was used to capture parents’ perceptions of the need for and usefulness of the manual, as well as their views of children’s worries. As such, content analyses were used by independent coders who determined codes, using memoing and notes over several reviews of the data to identify patterns, and then codes or themes in the data [27,28].

Coding Qualitative Data. Three coders conducted the content analysis (L.N., O.S., and A.R.) using an open coding process, reviewing transcripts, and using memos and notes to determine themes in the data [29,30]. L.N. and O.S. had experience with qualitative coding of interview data and content analysis. A third coder (A.R.) served as a verification coder [26]. First, coders reviewed data independently to determine categories or themes in the data. Then, coders met over three meetings (each 60 min) to develop and verify themes in the data as well as select representative quotes to support these themes. Disagreements were resolved by consensus.

Several processes established the trustworthiness of the qualitative methods [31]. Coders were experienced, and there were multiple reviews (individual and group meetings for peer debriefing and prolonged engagement with the data) of the data. Notes were taken at individual and group meetings to form an audit trail that could be used to discuss the process of discovering codes and themes in the data [32]. Moreover, having three coders, with one coder serving as a verification coder, ensured credibility, dependability, transferability, and confirmability within a rationalistic paradigm [31,33]. When coding the first set of interviews, there was saturation of themes in the data around interview ten, and similar themes were discovered in the second set of verification interviews [26,31]. Coders searched for negative cases, but none were observed. The iterative coding process, with rounds of coding, meetings, and confirming themes and representative quotes, improved the rigor of the coding process [31].

## 3. Results

### 3.1. Virtual Interviews with Parents

Demographics. Fifteen parents participated in Zoom interviews, two males and thirteen females. Parents provided data on age range, and 2 participants (13.3%) were in their 20s, 9 (60%) were in their 30s, 3 (20%) were in their 40s, and 1 (6.7%) was in her 50s. Six (40%) were white, 3 (20%) were African American, and 1 (6.7%) was Native American. Five parents (33%) did not wish to provide data on their racial group. Their children were in the first through fifth grades (all grades in this range were represented). Children were between the ages of 5 and 9 years (Mean = 7 years). There were 8 (53%) boys and 4 (27%) girls. Three of the parents (20%) did not provide information about the sex of their child. Ten of the parents provided data on the race of the child: six (40%) were white, 3 (20%) were African American, and 1 (7%) was Native American. Five (33%) of the fifteen parents did not wish to specify their racial group.

Themes for Parent Report. Five themes were discovered in the review of the data: (1) discussion of strategies parents would use with their child, (2) feeling that today’s children were worrying more often than children did in the past, (3) that the manual was useful, (4) new strategies that parents learned from reviewing the manual, and (5) alternative strategies used by parents beyond the manual.

Theme 1. Strategies Parents Would Use. The first theme was parents’ discussion of strategies they would use. Subthemes and some representative quotes for strategies parents would use are presented in Table 1. These strategies were children imagining their happy place, like vacations or parties; six parents used this technique. One of the parents shared,

“I often use the ‘beach’ example with my son when he tells me something is bothering him before bed, and I tell him to think about our vacations”. (Participant 1).

Another mentioned “She liked the imaginary thought or a cool place, going to your favorite place, like a party”. (Participant 5).

“The guided imagery strategy was a new strategy for me to use with my kids”. (Participant 15).

Beach ball breathing was commonly used by the parents (9). One of the parents mentioned, “So now, I will tell her to breathe whenever she is worried. I will just tell her to take a deep breath and that breathing in and breathing out can calm your nerves, you’ll be fine”. (Participant 7). Another also favored this: “I think I will use the beach ball breathing”. (Participant 6).

Additionally, three parents discussed using physical strategies such as the rock and sponge technique, which helped with relaxation. One of the parents said, “The one I like the most is the sponge, the sponge and the rock, you know there’s something about the opening and the closing your hands. I like the idea of the sponge squeezing out worry”. (Participant 3). Participant 7 shared, “The other one I found interesting was the rock and sponge.... rock is hard, so be very rigid, and then be a sponge, be very flexible, using the sponge to squeeze out whatever worry you have”.

Moreover, another three parents used the “Talking to Someone” strategy, emphasizing the value of encouraging children to “open up”. Participant 3 shared, “And I think that the strategies on talking to other people were also pretty good”. Another mentioned, “He was able to identify two people that he could talk to that he has access to” (Participant 9). “I think that it is very good for kids to talk about their worries in the classroom” (Participant 12).

Further, four parents favored using positive thinking as a strategy. “Thinking positively and using your imagination positively or be happier. That’s a good one to teach your child to think happily, think positively about something you like to do with family and friends. You don’t always have access to a puzzle or games so that’s a good one to use when we are not home”. (Participant 11). Another participant mentioned, “I think that thinking positively would be used, because I know that parents are using a lot of positives and like practicing positive affirmations with their children, which I like to see”. (Participant 12).

Lastly, using a distraction like toys or TV was used by two parents. “I know she’s always inclined towards reading books and playing with toys, she does pretend plays a lot... she can just come up with different characters and then start playing with it and that gets her to calm down” (Participant 7). Another parent mentioned, “Encourage her to color and read, I will begin to use distraction strategy more” (Participant 5).

Theme 2. What Children Worry About. The second theme reflected parents’ comments that today’s children were worrying more than in the past. Fourteen of the parents (93.3%) reported they felt ‘today’s children are worrying more. Table 2 presents what parents reported their children were worrying about. According to the interviews, eight parents thought their child worried about social media. One parent noted, “Well, I think they are worried about social media. Kids see other kids creating content, and now they are trying to meet up with a set standard” (Participant 7). Another shared, “They are exposed to a lot like Tiktok, Snap Chat, and they do a lot of comparing and it really hurts the kid’s self-esteem” (Participant 12).

Five parents reported children worry about getting into trouble with their parents or feel anxious when they think “Mom and Dad are mad at me”. One of the parents mentioned, “He does not like disapproval. If you communicate disapproval through body language or words, he’ll respond and be upset and cry” (Participant 3). Another mentioned, “What my child worries about the most is, is mommy upset with me? Mom is not happy with me” (Participant 7).

Three parents said their child worries about their safety and environmental concerns. “They spend a lot of their time thinking about safety” (Participant 4). Another mentioned, “I think kids worry a lot. I think kids worry about gun violence at school” (Participant 2).

Similarly, three other parents observed that their children worry about “parental concerns”, like finances and where their next meal will come from. One parent explained, “They are worried about their parents. If they have parents who are drug addicts, they could get taken away from their parents” (Participant 7). Another added, “Parents struggling financially makes children anxious about meals and home stability” (Participant 14).

Eight parents also reported that their child worries about their friends or has social concerns. One parent stated, ”There are things about school too they worry about. Like friends are not playing with me. I don’t know why she doesn’t want to play with me” (Participant 7). Another shared, “my kids worry a lot about social interactions, making friends, having friends... and that includes bullying and the larger idea about school safety” (Participant 5).

Lastly, eight parents stated that their children have academic concerns about school. “Some of my kids have a lot of anxiety about being picked on by the teacher in the classroom. They worry they won’t know the answer to a question or understand the content” (Participant 12). Another mentioned, “Children start to worry when they see that their classmates are getting it right, but they are not getting it right. They get worried at that moment” (Participant 10).

Theme 3. Usefulness of the Manual. The third theme focused on parents’ reasons why the manual would be useful for children (see Table 3). All the parents believed that the manual was useful for elementary school-aged children. According to the reports, five parents found the manual to be relatable for children. One parent stated, “I thought they were age-level appropriate. Overall, my son reads through it and comprehends the information” (Participant 9). Another shared, “I like the fact that it had things that are relatable, something that is easy for kids” (Participant 7).

Four parents mentioned it was a “Toolbox for parents”. One parent stated, “...unfortunately”. “I don’t think parents are given the kind of tools that you guys are creating here” (Participant 2). Another added, “Any material like this booklet is helpful at school or at home to get children to deal with their emotions” (Participant 11).

Six parents reported it was a “Good Resource” that was simple and manageable. One of the parents said, “The parents don’t need deep knowledge. Just very simple, practical things” (Participant 11). Another stated”, I love that it is short and direct. It doesn’t take a lot of time to work through. With book chapters, parents don’t have time to read it” (Participant 15).

Additionally, three parents said they like the variety of strategies in the manual. One parent explained, “I liked that there were a variety of strategies....you did a good job of offering variety of strategies” (Participant 4). Three parents described the manual as supporting emotional regulation. “It does a great job of helping kids refocus their minds on other things other than what is troubling them” (Participant 14).

Lastly, three parents said that it can be used at school or at home. One of the parents shared, “I think I will try to make copies for my school agers, because I want them to try this manual and read it themselves...and probably take it home and learn to calm themselves down” (Participant 8).

Theme 4. Strategies Learned from the Manual. The fourth theme focused on the new strategies parents learned after reviewing the worry strategy manual. Five parents (about 33% of the sample) said they learned a new strategy. Each parent endorsed one new strategy. One parent said they would use the beach ball breathing: “We enjoyed the breathing exercise. This is the beach ball breathing, sniffing the flowers and blowing up here” (Participant 8). One learned about positive thinking: “Thinking positively and using your imagination positively...that’s a good one to teach your child to think happy” (Participant 11). Another one found the distraction strategy helpful: “I think I wanna do the distraction one where you color a book or do a puzzle. I will start to use it soon, maybe next week when school starts” (Participant 14). One said they would use the sponge and rock breathing technique: “The one I like the most is the sponge, the sponge and the rock” (Participant 3). One mentioned using the imagination of a happy place with their children in the future: “The guided imagery strategy was a new strategy for me to use with my kids” (Participant 14). Notably, the parent who learned about distraction stated, “Alone time helps a child to process his or her emotions. So, I like setting (them) up with something like a puzzle to do” (Participant 11).

Theme 5. Use of Strategies Not in the Manual. The fifth theme reflected other strategies that parents mentioned using, specifically strategies they used that were not mentioned in the manual. There were 10 (66.7%) parents who discussed other strategies. Two parents used prayer or reading the Bible with their children as a calming strategy. “I am a Christian, so I will definitely pray with them” (Participant 6). Another mentioned, “The is relief in reading the word of God” (Participant 5). Two parents used drawing to help their child feel calm when he or she was worrying. One shared, “Drawing is a big part of what I do” (Participant 3). The other parent mentioned, “They can color as they read it” (Participant 6).

One parent discussed each of the following strategies: reading, yoga (including slow breathing), and journaling (journaling could include drawing and writing about worries). “I think about interactive manuals where children will be writing in it, I think about journaling and like we can add a space for children to write or draw things they might worry about” (Participant 9). One parent used counting as a calming strategy, stating, “Counting to calm down is a great technique, especially for younger kids. Using numbers as a way to calm down and focus helps children regulate emotions” (Participant 8). Another mother reported that she used to play with toys, often a favorite stuffed animal, to help their child calm down, “Using toys like their favorite stuffed animal or dolls to calm down works well” (Participant 5).

### 3.2. In-Person Interviews with Parents

Demographics. Six parents were interviewed, including four mothers (67%) and two fathers (33%). All were white. Age ranges for parents were as follows: 3 (50%) were 30–40, 2 (40%) were 40–50, and 1 (10%) was 51 and older. Their children were in kindergarten, first, second, fourth, and fifth grades. There were four (67%) girls and two (33%) boys. Five (83%) were white, and one (17%) was Asian. The children were 5, 6, 7, 8, 9, and 11 years (Mean = 7.7 years).

Theme 1: Strategies Parents Would Use. Parents stated they would try the following strategies with their child: talking to someone else (2 parents), breathing (3 parents), doing something fun (1 parent), superhero (1 parent), and thinking happy thoughts (1 parent). Three of the parents mentioned using more than one strategy with their child, including “breathing and thinking happy thoughts” (Participant 3, in-person interview), “breathing and the superhero” (Participant 5, in-person interview), and “breathing, doing something fun, and positive thoughts” (Participant 6, in-person interview). When asked if the parents would use some strategies for themselves, they mostly favored breathing exercises and talking to someone, with five parents preferring each. Three parents each mentioned they would apply “doing something fun” and “happy thoughts”, and one parent would use the rock sponge technique.

Theme 2. What Children Worry About. All the parents reported that their children experienced worry or anxiety. However, some reported that their child worried more frequently than others. Four reported their child worried “sometimes”, and two said their child worried “most of the time”. They reported that their children worried mostly about school (*n* = 5, such as about academic work). Three mentioned that their children worried about getting along with their friends or making friends. One parent stated her child’s worries were related to sensory-processing issues. All the parents reported that it was normal for children to worry.

Theme 3. Usefulness of the Manual. All parents viewed the manual as helpful for working with their children and as a tool they would use in the future to help their children cope with worry. Specifically, three said they would use it “sometimes”, and three said they would use it “all the time”. Only one of the parents offered comments for improving the manual, stating it was “a little academic in places” (Participant 1, in-person interview). She suggested continuing to work to make the explanation of worry and strategies more like a story and fun for children.

Theme 4. Strategies Learned from the Manual. Parents did not discuss learning new strategies from the manual but rather reported on what new strategies they would try with their child. Five of the parents reported they would now use multiple strategies (M = 3, range 1–5) with their child. One of the parents would only use one strategy (relaxation: turning one’s body into a rock and then a sponge, Participant 1, in-person interview). One parent planned to use “all of the strategies” (Participant 4, in-person interview). Happy thoughts and doing something fun were mentioned by three of the parents, and talking about it was mentioned by four of the parents. Having “multiple” strategies to use when their child was worried was an advantage of learning from the manual mentioned by several parents.

Theme 5. Use of Strategies Not in the Manual. In addition to the strategies from the manual, parents reported strategies they were already using, including fidget toys, art, drawing, shaping or playing with clay, and snuggles (hugs) One parent mentioned that her child’s sensory issues caused her to worry, and she used body movement with her child to “get her out of the worry loop” (Participant 1, in-person interview).

## 4. Discussion

This pilot study provided information about parents’ perceptions and ideas for using cognitive-behavioral strategies to help young children cope with anxiety in their everyday lives, providing critical information about strategies that parents and others, like teachers, can use with children to decrease feelings of anxiety. Parental perceptions of an anxiety management book for young children revealed their opinions of cognitive-behavioral anxiety management strategies and which ones they would implement with young children. The information provided by parents in online and in-person interviews was similar, indicating that the manual was useful and they would be able to use the strategies with their child. Although this study focused primarily on parents, its findings emphasized the feasibility of using the manual beyond the home setting. Some of the parents also affirmed its applicability in school settings. The strategies discussed in the manual are helpful for all caregivers, including teachers, counselors, and childcare providers, to assist children in managing anxiety across different environments.

Strategies Parents Would Use

Parents reported that cognitive-behavioral strategies, including breathing, relaxation, talking to someone else, positive imagery, and distraction, would help children reduce feelings of worry. Therefore, results were consistent with other studies showing that children benefit from using behavioral and cognitive strategies to reduce the experience of anxiety [12,14,15]. Parents reported that breathing techniques were a strategy they would use to help calm their children. Research has shown that these strategies help children calm themselves and promote relaxation [19]. Other strategies mentioned include positive thinking and distraction strategies, such as imagining a happy place with their children; these are core components of cognitive behavioral strategies that have been found effective in managing anxiety [17]. Adopting positive thinking and open conversations, as mentioned by the parents, would foster cognitive restructuring. This will help children reframe negative thoughts and feel better supported [20]. Integrating cognitive-based strategies into daily family routines may equip the children with early coping skills that promote emotional resilience before needing formal intervention [23]. Given the promising feasibility of using the manual among the parents in this study, it will be beneficial to assess its perceived usefulness among a larger and more diverse population. Future survey-based research may help strengthen the manual’s validation and broaden its applicability.

What Children Worry About

Parents indicated that their children were worrying about weighty real-world problems, like school success and family stresses, consistent with other research [3,4]. They believed that today’s children are worrying more than in the past and that social media could influence child anxiety [34]. Children were worrying about their academic performance, and things like their ability to speak in the classroom at school, and this may be one reason that parents mentioned the manual could be helpful in the school setting. Previous research indicated that teachers also thought the manual could help young children reduce anxiety related to academic performance and other worries while at school [25]. Research examining strategies to reduce anxiety about school performance will advance knowledge in the field.

Usefulness of the Manual

All parents, irrespective of the method of data collection, thought the manual was useful, had good information, and they stated they were going to implement cognitive-behavioral strategies from the manual with their children. Additionally, parents described the manual as easy to read, and thought strategies in the manual could be useful at home or as something that could be used at school if worries occurred in this setting. Studies have shown that active parents’ involvement in reinforcing cognitive-based strategies at home fosters early adoption and sustainability among children, creating a consistent environment for growth [22,24].

Strategies Learned from the Manual

Parents reported learning new techniques after reviewing the worry strategy manual, including beach ball breathing, positive thinking, guided imagery, and the sponge and rock technique that can be easily incorporated into daily life. This will allow parents to support their child’s emotional regulation and build resilience [22]. Many of the parents expressed enthusiasm about integrating the strategies learned into their routines.

Use of Strategies Not in the Manual

Parents were using some unique strategies not reviewed in the manual, such as music, prayer, and reading the Bible, drawing, playing with stuffies and fidget toys, as well as yoga and body movements. These strategies could be used to expand the manual. Moreover, it will be important to build upon parents’ “natural” or “typical” strategies so that they feel comfortable in expanding the interventions they use to help their child. Building parental skills and comfort in intervening with their child has the potential to reduce anxiety, which can build and become more serious in later childhood [9]. If parents have greater knowledge of how anxiety works and how to help their child, they may be able to help their child prevent it, and recognize when professional help is needed, which may reduce parental tendencies to avoid treatment for child anxiety [16].

We did not ask when strategies were used, and details on the situations in which children experienced anxiety and which strategies were used in different situations might provide direction for future research to determine strategies parents find most effective in moments of distress.

Limitations

This pilot study had several limitations, which may reduce the generalizability of study findings. For example, the sample sizes were small and were convenience samples, and interviewing more participants may lead to more commonalities in themes, especially regarding favorite strategies. However, results from the in-person interviews served as a type of verification check. The results of these interviews were consistent with those of the online interviews, showing that parents believed the manual was useful and identified strategies they would use with their children, which supported the purpose of developing the manual. A social desirability bias may have influenced parent reports during the interviews; however, they endorsed strategies they would not use, indicating they could provide negative responses. Researcher bias may have influenced coding; however, several verification procedures were used to determine themes in the data. Interviews were time-consuming, and future research using surveys, with items generated from the data from this study, may allow for a larger sample of parents to review the manual. Questions in the interviews did not examine how children’s worries evolved over time, and the interview itself occurred at one point in time. Longitudinal data on how children experience and cope with worry over time will provide information to guide interventions. As mentioned, interview questions did not assess how parents apply worry strategies in different situations, and learning whether different strategies work for different concerns will also inform intervention development. Parents were volunteers, and a positive bias may have predominated their response to the manual. The preponderance of participants were white, and obtaining more information from parents of color and learning more about how their own worries impact their own and their child’s lives will be important to address in future studies.

## 5. Conclusions

Training and equipping parents with developmentally appropriate, evidence-based strategies from the manual has the potential to improve children’s access to consistent support in managing anxiety at home. By providing early intervention, parents can foster emotional regulation, reduce anxiety symptoms, and build resilience over time among children. Additionally, recognizing that children respond differently to various techniques emphasizes the need for flexible, individualized approaches incorporated into the manual. As children engage in calming practices such as breathing exercises, guided imagery, or creative play, they internalize coping skills that may benefit them across developmental stages and in future anxiety-provoking situations. In the future, determining what strategies work for different stressors will be important. Moreover, learning how parents use strategies at home will provide information for developing programming that works for them. Also, learning about whether prevention efforts reduce the experience of anxiety in the long term will indicate whether prevention can reduce symptoms and decrease anxiety disorders in children and adolescents.

## Figures and Tables

**Table 1:** Strategies used by parents.

Subtheme	Number of Parents Endorsing Subtheme	Parent Perception of the Strategy (How Their Child Could Use It)
Imagination of happy place	6	“When he tells me something is bothering him before bed and tell him to think about our vacations” (Participant 1). “The imaginary thought or a cool place (going to your favorite place, like a party” (Participant 4).
Beach ball breathing	9	“A good one because you have to blow the air out. It can’t be like this it’s gotta be kind of a longer exhalation” (Participant 3). “I think the belly breath- the deep breathing is very important for the kids to learn how to calm themselves down” (Participant 12).
Rock and sponge	3	“They understand that sponges can soak and expel water so like there’s something about that visual I think that worked really well” (Participant 3).
Talking to someone	3	“He was able to identify two people that he could talk to that he has access to” (Participant 9).
Positive thinking	4	“Thinking happy thoughts or using imagination helps redirect anxiety. Imagining a happy place is a new technique I’ll try with my child” (Participant 11).
Distraction	2	“I know she’s always inclined towards reading books and playing with toys, she does pretend plays a lot... she can just come up with different characters and then start playing with it and that gets her to calm down” (Participant 7). “There is also watching TV or a movie or doing something fun. It is nice when it is just about something that you already know they like. It is not like a new thing entirely” (Participant 7).

**Table 2:** Things children are worrying about/what children are worrying about.

Subtheme	Number of Parents Who Endorsed Subtheme	Representative Quotes
Social media	8	“She wants to also have access to social media like her friends, that everyone has social media, everyone has Instagram. This social media is also affecting kids of this age” (Participant 7). “Like fitting in trends like social media and technology is really big... Meeting those trends and norms can cause a sense of worry in children” (Participant 9).
Are mom and dad mad at me	5	“So those are some things that makes her worried...Mommy is upset with me because of my behavior” (Participant 7).
Safety and environmental concerns	3	“She gave me this hug and said I have to hug you now because the earth is gonna break soon” (Participant 2). “Kids worry about gun violence” (Participant 4). “You can’t sugarcoat the fact that no matter what language you use, you’re having children do a drill in case someone tries to come in and put a bullet in their head” (Participant 2).
Parental stresses	3	“Parents struggling financially makes children anxious about meals and home stability” (Participant 14). “Mostly, I think he’s worried about not going to the swimming pool. I don’t swim. I think they are worried about me not being able to swim” (Participant 6). “They are worried about their parents. If they have parents who are drug addicts, they could get taken away from their parents” (Participant 7).
Friends/social concerns/bullying	8	“Worried that his friends won’t like him....friendship pressure people will be friends with them one day and then not friends with him the next” (Participant 3). “She worried about making friends are her new school when we moved and bullying it happened the first few weeks in her new school” (Participant 5).
School/academic achievement concerns	8	“When they are in school, and they are given schoolwork to do, and they are having difficulties in getting it done”. (Participant 10) “They worry about grades a lot. My 3rd grader worries about workload and keeping track of schoolwork, it causes him a lot of stress” (Participant 4). “He’s already anxious about the sounds that he makes the way that he misuses the language or the way that he’s learning to use it” (Participant 3).

**Table 3:** Parents’ reasons for why the worry strategy manual was useful for children.

Reason	Number of Parent Endorsements	Representative Quotes
Relatable for children	5	“I thought the strategies were good ideas that kids could relate to” (Participant 1).
Toolbox for parents	4	“I didn’t know this type of book exists. I feel I have a good start and I feel assured... I have learned some new things that I can add to what I already know to help my child” (Participant 6).
Good resource: Simple and manageable	6	“I thought it was really nice. It was a very simple and manageable group of skills” (Participant 4).
Variety of strategies	3	“It provides multiple strategies, allowing children to choose what works best”. “That’s what I like about the booklet; it gives you a handful of practices, and you can figure out which is most successful” (Participant 3).
Encourages emotional regulation	3	“The booklet helps children understand and regulate their emotions” (Participant 11). “Having the opportunity to teach and reinforce these skills helps the whole classroom” (Participant 4).
Can be used at school and at home	3	“The strategies are applicable both at home and in the classroom. It is very good for kids to talk about their worries in the classroom” (Participant 12)

## Data Availability

Data are available from the authors.
